# LC-Q-Orbitrap-MS/MS Characterization, Antioxidant Activity, and α-Glucosidase-Inhibiting Activity With *In Silico* Analysis of Extract From *Clausena Indica* (Datz.) Oliv Fruit Pericarps

**DOI:** 10.3389/fnut.2021.727087

**Published:** 2021-09-01

**Authors:** Ruimin Wang, Ruiping He, Zhaohui Li, Lu Wang

**Affiliations:** ^1^School of Food Science and Engineering, Hainan University, Haikou, China; ^2^Key Laboratory of Food Nutrition and Functional Food, Hainan University, Haikou, China

**Keywords:** *Clausena indica* (Datz.) Oliv fruit pericarps, phenolic compounds, biological properties, *in silico* analysis, multivariate analysis

## Abstract

*Clausena indica* (Datz.) Oliv fruit pericarps (CIOPs) is an important agro-industrial by-product rich in active components. In this article, the effects of traditional and green deep eutectic solvents (DESs) on the high-performance liquid chromatography (HPLC) characterization, antioxidant activities, and α-glucosidase-inhibitory activity of phenolic extracts from CIOPs were investigated for the first time. The results showed that ChCl-Gly and Bet-CA had higher extraction efficiency for the total phenolic content (TPC, 64.14–64.83 mg GAE/g DW) and total flavonoid content (TFC, 47.83–48.11 mg RE/g DW) compared with the traditional solvents (water, methanol, and ethyl acetate). LC-Q-Orbitrap-MS/MS was adopted to identify the phenolic compositions of the CIOPs extracts. HPLC-diode array detection (HPLC-DAD) results indicated that arbutin, (–)-epigallocatechin, chlorogenic acid, procyanidin B1, (+)-catechin, and (–)-epicatechin were the major components for all extracts, especially for deep eutectic solvents (DESs). In addition, ChCl-Xyl and ChCl-Gly extracts showed higher antioxidant activities against 2,2-diphenyl-1-picrylhydrazyl (DPPH^•^), 2,2′-azino-bis (3-ethylbenzothiazoline-6-sulphonic acid (ABTS^+•^), ferric reducing antioxidant power (FRAP), reducing power (RP), and cupric ion reducing antioxidant capacity (CUPRAC) than extracts extracted by other solvents. A strong α-glucosidase-inhibiting activity (IC_50_, 156.25-291.11 μg/ml) was found in three DESs extracts. Furthermore, *in silico* analysis of the major phenolics in the CIOPs extracts was carried out to explore their interactions with α-glucosidase. Multivariate analysis was carried out to determine the key factors affecting the antioxidant activity and α-glucosidase-inhibiting activity. In short, DES can be taken as a promising solvent for valorization and recovery of bioactive compounds from agro-industrial by-products. The results verified that CIOPs can be used as a prospective source rich in bio-active compounds applied in the food and pharmacy industries.

## Introduction

*Clausena indica* (Datz.) Oliv, which belongs to the wild evergreen arbor plant of the Rutaceae family, is mainly distributed in the Southern and Southeastern Asian countries (1). In China, it is mainly distributed in Hainan Island, Guangxi, and Yunnan, etc. The fruit of *C. indica* is commonly known as “Jipi fruit,” a rare and special sour-sweet berry with an intense aroma that can be used as a type of traditional Chinese medicine as well. In the folk, *C. indica* fruit is widely used for strengthening the spleen and improving human immunity (2). Many researchers have confirmed that *C. indica* fruit is rich in polyphenols, coumarins, alkaloids, terpenoids, and has various biological activities, such as anti-oxidation, anti-diabetics, anti-bacteria, anti-inflammation, lipid-modifying, and liver-protective effects (3). At present, the processed products of *C. indica* fruit mainly include beverage, jam, and preserved fruit, but a large amount of *C. indica* fruit pericarps (CIOPs) are often discarded as waste in the process of industrial processing, causing considerable environmental pollution. Therefore, it is of paramount importance to realize the valorization and recovery of bioactive compounds from CIOPs.

In general, recovering compounds from the agro-industrial by-products or plant matrix is performed using organic solvents. Although organic solvents extract bio-active components, they have inevitable shortcomings, such as toxicity, non-degradability, low boiling points, and high flammability (4–8). Currently, deep eutectic solvents (DESs) are synthesized with hydrogen bond acceptors (HBAs) and hydrogen bond donors (HBDs) under a low melting temperature. DESs are favored for the simple preparation process, lower synthesis cost, biodegradability, negligible volatility, non-flammability, favorable stability, and renewability (5, 9–11). It is particularly worth mentioning that DESs can badly damage the structure of plant cell walls by dissolving lignocellulose and lignin and thereby significantly increasing the extraction yields of bioactive components from the agro-industrial by-products (12, 13). de Almeida Pontes et al. (2021) have reported that DESs are innovative, environmentally friendly, and high-performance solvents for extracting the phenolic compounds from olive leaves (14). Shang et al. (2019) adopted 20 types of DESs to extract isoflavones from chickpea sprouts and found that a mixture of choline chloride and propylene glycol (1:1, *mol*/*mol*) showed excellent extraction efficiency for isoflavone compounds (15). Marcos et al. (2020) found that a tailor-made eutectic solvent yielded the highest contents of phenolic compounds, anthocyanins, total sugars, and acid sugars from strawberry or raspberry, and the extract also presented better antioxidant activity (12). To date, there are few reports on the chemical characterization and green valorization of bio-active compounds from CIOPs.

This study aimed to systematically investigate the impacts of conventional and greenly solvents on the high-performance liquid chromatography (HPLC) characterizations, antioxidant activities, and α-glucosidase-inhibiting activity of phenolic extracts from CIOPs. The phenolic compositions of the CIOPs extracts were identified and quantified by LC-Q-Orbitrap-MS/MS for the first time. *In silico* analysis was carried out to investigate the binding mechanisms of major phenolic compounds to α-glucosidase. A multivariate analysis was performed to determine the main contributors to the antioxidant activity and α-glucosidase-inhibiting activity of the CIOPs extracts. This study may provide important evidence for the valorization and utilization of *C. indica* fruit pericarps.

## Materials and Methods

### Materials and Chemicals

*Clausena indica* (Datz.) Oliv fruit was collected from Wuzhishan City of Hainan Province, China. *C. indica* (Datz.) Oliv fruit pericarps (CIOPs) were dried in a vacuum freeze drier (Songyuan Huaxing LGJ-12, Beijing Songyuan huaxing Technology Develop Co., Ltd, Beijing, China), pulverized by a disintegrator, sieved through a 60 mesh, and finally stored hermetically at 4°C until use. α-Glucosidase from *Saccharomyces cerevisiae* and 4-N-trophenyl-α-D-glucopyranoside (*p*-NPG) were purchased from Sigma Chemical Co., Ltd. (Shanghai, China). All of the phenolics standards, Folin-Ciocalteu's phenol reagent, 6-hydroxy-2,5,7,8-tetramethylchroman-2-carboxylic acid (Trolox), 1,1-diphenyl-2-picrylhydrazyl (DPPH^•^), 2, 2'-azino-bis (3-ethylbenzothiazoline-6-sulfonic acid) diammonium salt (ABTS^+•^), 2,4,6-tripyridyl-s-triazine (TPTZ), and all chemicals used to prepare DESs were obtained from Aladdin Biochemical Technology Co. Ltd. (Shanghai, China). Formic acid, methanol, and acetonitrile for HPLC analysis were obtained from Fisher Scientific (MA, USA). Na_2_CO_3_, NaNO_2_, AlCl_3_, methanol (MeOH), and ethyl acetate (EtAc) were purchased from Nanjing Chemical Engineering Factory (Nanjing, China).

### Preparation of DESs

Deep eutectic solvents were synthesized according to the procedure described in the previous study (16). The starting components were added to a flask with a suitable molar ratio and heated at 80°C until the formation of transparent and homogeneous liquid. Then, 30% ultra-pure water (w/w) was added to reduce the DES viscosity for subsequent extraction. Table 1 shows the information of the starting components for DESs preparation.

**Table 1:** List of DESs prepared in this study.

**No**.	**Abbreviation**	**Component A**	**Component B**	**Component C**	**Molar ratio (mol/mol)**
1	ChCl-MA	Choline chloride	Malic acid	–	1:1
2	ChCl-Prop	Choline chloride	1,2-Propanediol	–	1:2
3	ChCl-Glu	Choline chloride	Glucose	–	5:2
4	ChCl-Xyl	Choline chloride	Xylitol	–	1:1
5	ChCl-LA	Choline chloride	Lactic acid	–	1:2
6	ChCl-Gly	Choline chloride	Glycerol	–	1:2
7	ChCl-LevA	Choline chloride	Levulinic acid	–	1:2
8	ChCl-Oxa	Choline chloride	Oxalic acid	–	1:1
9	ChCl-MetA	Choline chloride	Methanoic acid	–	1:1
10	ChCl-MA-Xyl	Choline chloride	Malic acid	Xylitol	1:1:1
11	ChCl-MA-Pro	Choline chloride	Malic acid	L-Proline	1:1:1
12	ChCl-Urea-Prop	Choline chloride	Urea	1,2-Propanediol	1:1:1
13	Bet-Gly	Betaine	Glycerol	–	1:1
14	Bet-CA	Betaine	Citric acid	–	1:1
15	Bet-LA	Betaine	Lactic acid	–	1:2
16	Pro-Gly	L-Proline	Glycerol	–	1:2
17	Pro-EthG	L-Proline	Ethylene glycol	–	1:2
18	LA-Glu	Lactic acid	Glucose	–	1:1.23

### Extraction of Phenolic Compounds From CIOPs

*Clausena indica* (Datz.) Oliv fruit pericarps powder (0.5 g) was mixed with 5 ml of extraction solvents (H_2_O, 50% MeOH, EtAc, and 18 types of DESs) in 10 ml tubes, respectively. The extraction procedure was performed in an ultrasonic bath at 320 W, 40°C for 30 min, followed by centrifugation at 10,000 × *g* for 5 min to collect supernatants.

### Determination of Total Phenolic Content (TPC) and Total Flavonoid Content (TFC)

Total phenolic content (TPC) in the CIOPs extracts was determined with the colorimetric Folin–Ciocalteu method (17). Briefly, 20 μl of the CIOPs extracts were incubated with 200 μl of Folin–Ciocalteu reagent at 25°C for 5 min, followed by the addition of 400 μl of saturated Na_2_CO_3_ solution for another 30 min of incubation, and then, the absorbance was tested at 765 nm. Data were denoted as milligrams of gallic acid equivalents (GAE) per gram dry weight (DW) of CIOPs (mg GAE/g DW). The calibration curve of gallic acid (Y = 0.0032 X – 0.0004, R^2^ = 0.996) was drawn. Total flavonoid content (TFC) was tested with the aluminum chloride method (18, 19). Let 100 μl of extract react with 50 μl of 5% NaNO_2_ (*w/v*) for 5 min and then, add 50 μl of 10% AlCl_3_ (*w/v*) for another 6 min of reaction. Finally, 400 μl of 1 M NaOH and 400 μl of water were mixed and incubated for 15 min before reading the absorbance at 510 nm. The calibration curve for rutin (Y = 0.0006 X – 0.0143, R^2^ = 0.997) was drawn. Data were denoted as milligrams of rutin equivalents (RE) per gram dry weight (DW) of CIOPs (mg RE/g DW).

### Identification and Quantification of Phenolic Compositions

All the extracts were subjected to a 0.22 μm filter before being analyzed. The identification of phenolic compounds was performed by using an Agilent 1,260 HPLC system equipped with a DAD detector (Agilent Technology, Santa Clara, CA, USA), a reversed-phase Aligent Zorbax SB C18 plus column (250 mm × 4.6 mm, 3.5 um), and a Q Exactive HFX mass spectrometer (Orbitrap MS, Thermo Fisher Scientific, Waltham, MA, USA). The mobile phase was composed of phase A (0.1% formic acid in acetonitrile) and phase B (0.1% formic acid in water). The constant flow rate was 0.8 ml/min, the injection volume was 10 μl, and gradient elution conditions were as follow: 0–5 min, 15% A; 5–25 min, 25–35% A; 25–40 min, 35–50% A; 40–45 min, 85% A; and 45–50 min, 15% A. The QE HFX mass spectrometer was adopted to acquire tandem mass spectrometry (MS/MS) on the information-dependent acquisition (IDA) mode in the control of the acquisition software (Xcalibur, Thermo Fisher Scientific, Waltham, MA, USA). In this mode, the acquisition software continuously evaluates the full scan MS spectrum. The ESI source conditions included 30 Arb of sheath gas flow rate, 10 Arb of aux gas flow rate, 350°C of capillary temperature, 60,000 of full MS resolution, collision energy as 10/30/60 in NCE mode, 4.0 kV (positive) or −3.8 kV (negative) of spray voltage, respectively. The acquired MS data were processed using Bruker Daltonics Data Analysis software. The chromatographic conditions for HPLC-DAD quantification analysis were referred to in the previous work (17). A linear standard curve was plotted using series dilutions of standards with known concentrations (Supplementary Table 1). The contents of the analytes were expressed as milligram per gram dry weight (mg/g DW).

### Evaluation of Antioxidant Activities *In vitro*

Antioxidant activities *in vitro* of the CIOPs extracts were determined using five methods, such as DPPH^•^, ABTS^+•^, FRAP, RP, and CUPRAC assay. The DPPH^•^ and ABTS^+•^ radical-scavenging activity of the CIOPs extracts were determined using the method proposed by Wu et al. (20). CUPRAC assay was performed according to the procedure reported by Wang et al. (21). The reported methods of Wang et al. (17) were used to measure FRAP and RP values of the CIOPs extracts. The results of DPPH^•^, ABTS^+•^, RP, and CUPRAC were denoted as micromoles of Trolox equivalents (TE)/g DW, furthermore, the FRAP values were denoted as micromoles Fe^2+^ equivalents of Fe(II)E/g DW.

### Determination of α-Glucosidase Inhibiting Activity (α-GIA)

The α*-*glucosidase inhibiting activity (α*-*GIA) was measured following the procedures described in the previous study (22), with slight modifications. Briefly, 100 μl 0.5 U/ml α-glucosidase dissolved in 0.1 M phosphate buffer (PBS, pH 6.8) was incubated with 100 μl of the extracts or individual phenolics at 37°C for 10 min, followed by the addition of 100 μl of 5 mM *p*-NPG solution for 20 min of reaction at 37°C, and then the addition of 500 μl of 1 M Na_2_CO_3_ solution for termination. The absorbance was measured at 405 nm. The α*-*GIA of the extracts was repressed as half inhibit concentration (IC_50_) value. The α-GIA was calculated based on the following equation using acarbose and PBS as the positive and negative control, respectively:

$$\begin{array}{l} \text{α}-\text{GIA}\left(\text{\%}\right)=\left[\left({\text{A}}_{0}-{\text{A}}_{1}\right)/{\text{A}}_{0}\right]*\;100\text{\%}\\ \text{where Δ}{\text{A}}_{0}={\text{A}}_{\text{PBS}+\text{enzyme}}-{\text{A}}_{\text{PBS}},\text{Δ}{\text{A}}_{1}\\ \;=A_{\text{extract}+\text{enzyme}}-{\text{A}}_{\text{extract}} \end{array}$$

### *In silico* Analysis

To provide deep insight into the interaction between the main phenolics and α*-*glucosidase, an *in silico* docking study in Surflex-Dock Geom (SFXC) mode was performed using SYBYL-X 2.0 software (Tripos, Inc., St. Louis, MO, USA). The 2D structures of the main phenolics and acarbose were plotted using ChemBio3D Ultra software (MA, USA). The homologous structure of α*-*glucosidase (PDB ID: 3A4A) was obtained from the RCSB Protein Data Bank (RCSB PDB). Acarbose, as a well-known inhibitor for α*-*glucosidase, was adopted as the positive control. After docking with α*-*glucosidase, the key parameters of the docking of the ligand with α-glucosidase were generated. Based on the previous reports, a C-score ≥ of 4 was deemed as a credible docking result. T-score value, as a weighted sum of non-linear functions, indicates van der Waals surface distance of the interaction of the ligand with α-glucosidase in docking analysis. The number and distances of hydrogen bonds and residues of amino acid active sites may clarify the interactive effects between the main phenolics and α-glucosidase (23).

### Statistical Analysis

All results were measured three times and denoted as the mean and SD values (mean ± SD). The IC_50_ value was obtained *via* Probit analysis. The multivariate analysis and statistical analysis were carried out by SPSS v26.0 software using one-way One Factor ANOVA. The differences were significant at a level of *p* < 0.05.

## Results and Discussion

### Total Phenolic Content and Total Flavonoid Content

**Figure 1:**
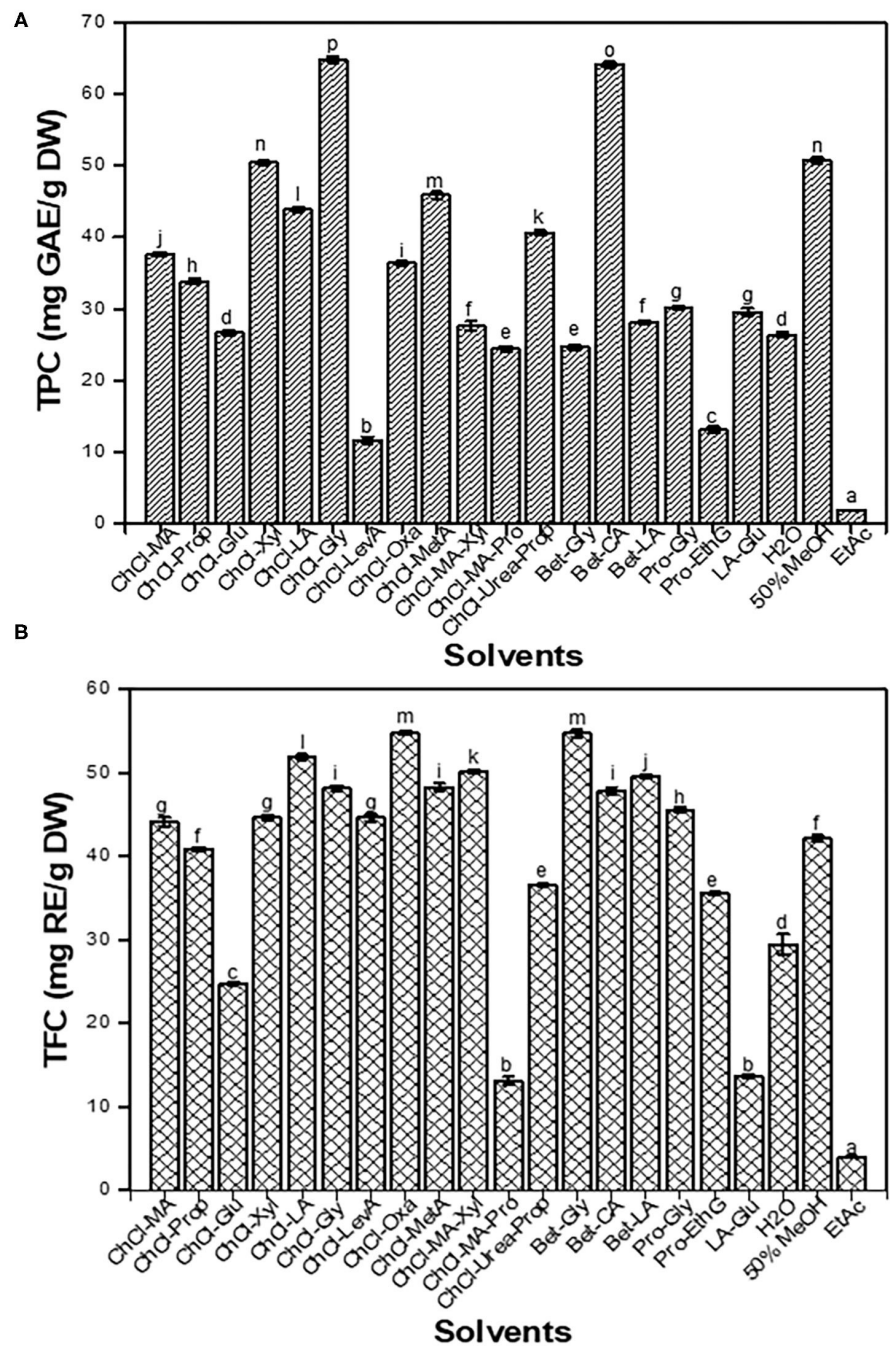
Total phenolic content (TPC) **(A)** and total flavonoid content (TFC) **(B)** of the *Clausena indica* (Datz.) Oliv fruit pericarps (CIOPs) extracts were obtained by the traditional solvents and deep eutectic solvents (DESs). Different lowercase letters (a–m) mean statistically significant differences in TPC/TFC extracted with different solvents.

Deep eutectic solvents, as a mixture of different types of HBDs and HBAs, have different physicochemical properties, such as viscosity, polarity, pH, and solubility. Consequently, DESs greatly affect the extraction efficiency for bio-active components from agro-industrial by-products (24). In this work, the traditional solvents and 18 types of DESs were used to extract TPC and TFC from CIOPs, and their extraction efficiencies were analyzed (Figures 1A,B). It was found that ChCl-Xyl and ChCl-Gly were more effective in extracting TPC (64.14–64.83 mg GAE/g DW) than other DESs. Intriguingly, except ChCl-Glu, ChCl-MA-Pro, and LA-Glu (13.05–24.72 mg RE/g DW, respectively), other DESs had high efficiency in the extraction of TFC. Regarding the traditional solvents, we found that 50% MeOH extract showed the highest TPC (50.75 mg GAE/g DW), followed by water (26.38 mg GAE/g DW) extracts. EtAc extract had the lowest TPC (1.84 mg GAE/g DW) and TFC (3.93 mg RE/g DW). It was observed that acidic- or polyalcohol-based DESs had higher extraction efficiencies for phenolics/flavonoids compounds from CIOPs than amide- and sugar-based DESs did, which was consistent with the results of the previous studies (24, 25). As we know, the viscosity of DES is a critical factor affecting solid-liquid extraction. Excessively high viscosity greatly influenced mass- and energy-transfer, thereby affecting the extraction efficiency of active compounds from agro-industrial by-products. The viscosities of sugar-based DESs were significantly higher than those of other types of DESs (26, 27). In addition, the polarity of the solvents also affected the extraction efficiency of phenolic compounds significantly. DESs with a wide range of polarity were reported to have high efficiency in the extraction of bio-active compounds from natural products (28, 29). Taken together, ChCl-Xyl, ChCl-Gly, and Bet-CA could remarkably enhance the extraction of TPC and TFC from CIOPs, so they were chosen as the extraction solvents, and their compositions and bio-activities were comparatively analyzed. Sarikurkcu et al. (30) determined the yields of total phenolic and flavonoids from *Onosma pulchra* by using EtAc, MeOH, and water as the extraction solvents, and verified EtAc had the lowest extraction efficiency, which was in line with the result of this study. Zhu et al. (16) investigated the effects of different solvents (water, organic solvents, and DESs) on the extraction of phenolic compounds from *Morinda citrifolia* L. leaves, and found that DESs extracts yielded higher contents of phenolic compounds and had stronger biological activities, which was in accordance with the results of the current studies.

### Phenolic Compositions

**Figure 2:**
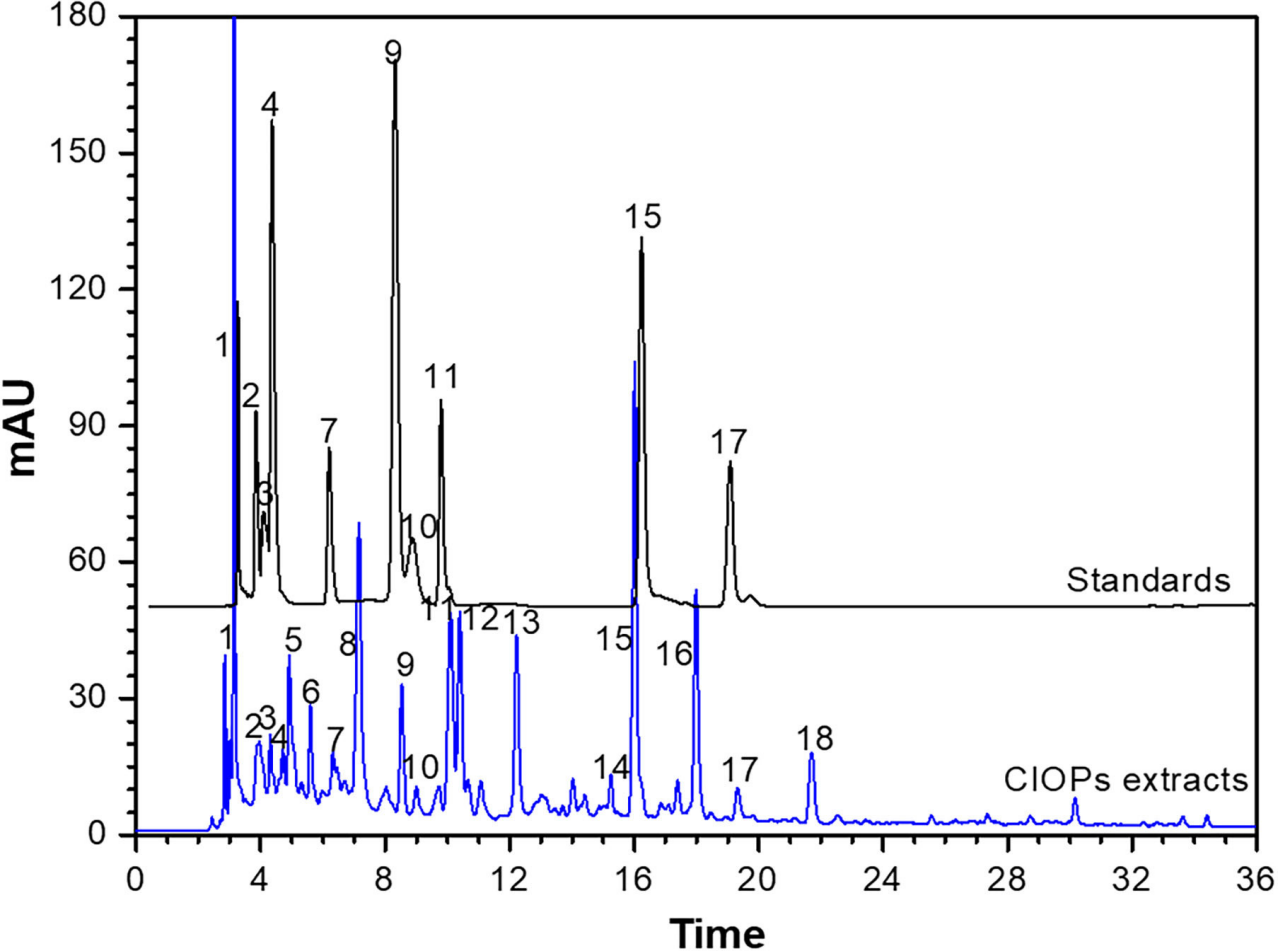
High-performance liquid chromatography (HPLC) profiles of the phenolic compounds of the CIOPs extracts and the standards.

As shown in Table 2 and Figure 2, identification of chemical constituents in the CIOPs extracts was implemented by comparing retention time (RT), mass fragmentation pattern, and accurate mass information with public databases and the standards.

**Table 2:** Identification of the main phenolic compounds from CIOPs extracts.

**No**.	**Retention time (min)**	****λ_max_ (nm)****	**Molecular ion (*m/z*)**	**MS ion fraction (*m/z*)**	**Mw**	**Formula**	**Compounds**	**Error**	**Reference**
1	2.91	280, 360	271.10 [M-H]^−^	271.10, 143.01, 125.10	272	C_12_H_16_O_7_	Arbutin	1.1	Standard, MS/MS
2	3.60	256, 350	305.06 [M-H]^−^	305.10, 219.10, 177.01	306	C_15_H_14_O_7_	(–)-Epigallocatechin	−0.2	Standard, MS/MS
3	3.64	254, 280	353.09 [M-H]^−^	353.09, 191.06, 185.05	354	C_10_H_10_O_4_	Chlorogenic acid	0.3	Standard, MS/MS
4	3.69	350, 542	577.15 [M-H]^−^	577.15, 407.05, 303.01, 289.01	578	C_30_H_26_O_12_	Procyanidin B1	1.2	Standard, MS/MS^31^
5	4.87	254, 280	410.16 [M-H]^−^	455.16, 410.16, 354.36	411	C_20_H_26_O_9_	5-O-Caffeoylquinic acid butyl ester	−0.4	MS/MS
6	5.62	254, 280	352.15 [M-H]^−^	352.15, 351.15	353	C_18_H_24_O_7_	Renifolin	−0.7	MS/MS
7	6.21	260, 350	289.07 [M-H]^−^	289.07, 245.08	290	C_15_H_14_O_6_	(+)-Catechin	−0.5	Standard, MS/MS
8	7.15	280, 350	451.32 [M-H]^−^	451.32, 289.07, 245.08, 161.03	452	C_21_H_24_O_11_	Catechin 7-O-β-D-glucopyranoside	2.7	MS/MS
9	8.63	254, 280	153.02 [M-H]^−^	153.02, 108.02	154	C_7_H_6_O_4_	Protocatechuic acid	0.9	Standard, MS/MS
10	8.92	256, 350	289.07 [M-H]^+^	289.07, 245.08	290	C_15_H_14_O_6_	(–)-Epicatechin	−0.3	Standard, MS/MS^32^
11	10.07	214, 280	179.04 [M-H]^−^	179.04, 135.05	180	C_9_H_8_O_4_	Caffeic acid	0.6	Standard, MS/MS^32^
12	10.25	280, 350	565.15 [M+H]^+^	609.15, 565.15, 563.13, 404.09, 270.24, 161.13	564	C_26_H_28_O_14_	Apigenin-6-arabinose-8-glucose	1.8	MS/MS
13	12.17	280, 350	461.12 [M-H]^−^	462.12, 461.12, 301.21, 151.07	462	C_22_H_22_O_11_	unknown	4.2	–
14	15.21	280, 350	448.10 [M-H]^−^	448.10, 286.15, 161.37	449	C_21_H_20_O_11_	Kaempferol-3-O-β-D-glucopyranoside	0.9	MS/MS
15	15.95	254, 280	151.03 [M-H]^−^	136.00,106.90, 97.50	152	C_8_H_8_O_3_	Vanillin	−0.4	Standard, MS/MS
16	17.87	280, 354	491.10 [M-H]^−^	492.12, 491.10, 301.21, 161.03	492	C_23_H_24_O_12_	unknown	4.7	–
17	19.19	260, 350	303.25 [M-H]^−^	303.25, 301.02	304	C_15_H_12_O_7_	Taxifolin	−0.1	Standard, MS/MS
18	21.68	254, 280	189.05 [M-H]^−^	190.05, 189.05	190	C_11_H_10_O_3_	unknown	−0.8	–

Compound 1 (RT 2.91 min), showing molecular ion 271.10 [M-H]^−^ with MS ion fractions at *m/z* 271.10, 125.10, and 143.01 and molecular formula C_12_H_16_O_7_ (λ_max_ around at 280 and 360) was identified as arbutin. Compound 2 (RT 3.60 min), which was an isomer of catechin derivatives exhibiting parent ion *m/z* of 305.06 [C_15_H_14_O_7_-H]^−^, was identified as (–)-epigallocatechin. Compound 4 (RT 3.69 min) with the fragment ions at *m/z* 577.15 [2C_15_H_14_O_6_-H]^−^, 407.05, 303.01, and 289.07 [C_15_H_14_O_6_-H]^−^ were identified as major fragment ions of B-type proanthocyanidins (procyanidin B1) (31). Compound 5 with the parent ion at *m/z* 410.16 [C_20_H_26_O_9_-H]^−^ and the fragment ion at *m/z* 354.36 [C_16_H_18_O_9_-H]^−^ was temporarily regarded as 5-*O*-caffeoylquinic acid butyl ester. Compound 6, indicating the parent ion at *m/z* 352.15 [C_18_H_24_O_7_-H]^−^, was temporarily determined as renifolin. Three phenolic acids (compound 3, 9, and 11) were easily determined as chlorogenic acid (RT 3.64 min, [M–H]^−^ ion at *m/z* 305), protocatechuic acid (RT 7.92 min, [M–H]^−^ ion at *m/z* 153.02), and caffeic acid (RT 9.57 min, [M–H]^−^ ion at *m/z* 179.04), respectively (32). Compounds 7 and 10 had the same parent ion at *m/z* 289.07 [C_15_H_10_O_6_-H]^−^ and ion fraction at *m/z* of 245.08, which were identified as (+)-catechin and (–)-epicatechin by comparing the retention time with standards, respectively. Compound 8 with the molecular ion at *m/z* 451.32 [M-H]^−^ and the fragment ions at *m/z* 289.07 [C_15_H_10_O_6_-H]^−^ and *m/z* 161.03 [M-C_15_H_10_O_6_-H]^−^ was temporarily identified as catechin 7-O-β-D-glucopyranoside. Compound 12 (RT 10.25 min, λ_max_ around at 280 and 350) was likely to be apigenin-6-arabinose-8-glucose considering the molecular ion at *m/z* 565.15 [C_26_H_28_O_14_-H]^−^ with MS ion fraction at *m/z* 404.09 [C_26_H_28_O_14_-glc-H]^−^ *m/z* 270.24 [C_15_H_10_O_5_-H]^−^, and 161.13 [Glc-H]^−^. Compound 14, having the parent ion at *m/z* 448.10 [C_21_H_20_O_11_-H]^−^ and fragment ions at *m/z* 286.15 [C_15_H_10_O_6_-H]^−^ and *m/z* 161.37 [M-C_15_H_10_O_6_-H]^−^, was regarded as kaempferol-3-O-β-D-glucopyranoside. Compound 15 (RT 15.95 min) showing the molecular ion at *m/z* 151.03 [C_8_H_8_O_3_-H]^−^ was assigned to vanillin. Compound 17 (RT 19.19 min) with the molecular formula of C_15_H_12_O_7_, the molecular ion at *m/z* 303.25 [M-H]^−^, and fragment ions at *m/z* 303.25 and 301.02, was identified as taxifolin, which was consistent with previous literature (33). Compounds 13, 16, and 18 cannot be temporarily identified according to the current information.

According to data given in Table 3, 10 identified phenolic compounds from the CIOPs extracts were quantified by commercial standards (Supplementary Table 1). Three DES extracts showed high contents of individual phenolics, such as arbutin (0.80 mg/g DW in ChCl-Xyl), (–)-epicatechin (0.72 mg/g DW in ChCl-Xyl), (–)-epigallocatechin (11.43 mg/g DW in Bet-CA), chlorogenic acid (2.70 mg/g DW in ChCl-Gly), procyanidin B1 (2.35 mg/g DW in ChCl-Gly), (+)-catechin (3.41 mg/g DW in ChCl-Gly), and taxifolin (0.42 mg/g DW in ChCl-Gly). Protocatechuic acid, caffeic acid, and taxifolin with relatively low contents can be detected in the extracts extracted with traditional solvents. In addition, taxifolin can be only detected in the extracts extracted using water, 50% MeOH, and ChCl-Gly. Vanillin was only detected in the three DESs extracts. Regarding the extracts extracted using traditional solvents, water, and 50% MeOH extracts showed high contents of (–)-epigallocatechin, chlorogenic acid, procyanidin B1, and (+)-catechin. Only four phenolic compounds [(–)-epigallocatechin, chlorogenic acid, procyanidin B1, (–)-epicatechin] with the lowest contents can be detected in the EtAc extract. Meanwhile, all identified phenolic compounds existed in water extract, except for vanillin, as compared to extracts by ChCl-Xyl, ChCl-Gly, and Bet-CA. The present work confirmed that extraction solvents significantly affected the contents of phenolic compounds in the CIOPs extracts (34, 35). Simultaneously, eco-friendly DESs exhibited higher efficiency in extracting the individual phenolic compounds from CIOPs than traditional solvents, so they can be used for valorization and recovery of special high-value compounds from agro-industrial by-products.

**Table 3:** The contents of the main phenolic compounds of the CIOPs extracts with different solvents.

**Contents (μg/g DW)**	**Extractions**					
	**Water**	**50% MeOH**	**EtAC**	**ChCl-Xyl**	**ChCl-Gly**	**Bet-CA**
Arbutin	216.05 ± 14.75a	622.58 ± 30.40c	ND	803.18 ± 9.09e	765.49 ± 5.41d	499.30 ± 12.51b
(–)-Epigallocatechin	4,525.25 ± 63.29c	815.04 ± 52.41b	164.73 ± 23.24a	768.64 ± 13.52b	811.77 ± 16.30b	11,430.14 ± 184.92d
Chlorogenic acid	1,144.76 ± 1.94b	1272.96 ± 49.65c	72.26 ± 2.42a	2,335.15 ± 26.58d	2,702.66 ± 14.40e	2,393.88 ± 15.35d
Procyanidin B1	430.91 ± 13.58b	ND	326.75 ± 3.19a	2,323.24 ± 3.19d	2,345.26 ± 24.65d	1,671.78 ± 25.44c
(+)-Catechin	1,005.77 ± 6.23a	1,311.11 ± 49.88b	ND	3,058.23 ± 29.46d	3,410.49 ± 76.88e	2,300.66 ± 10.31c
Protocatechuic acid	13.76 ± 0.13a	15.89 ± 1.03b	ND	ND	ND	ND
(–)-Epicatechin	147.64 ± 2.00b	268.47 ± 5.23c	85.85 ± 2.46a	720.27 ± 13.47e	699.98 ± 4.11e	483.37 ± 12.11d
Caffeic acid	36.69 ± 0.83a	50.29 ± 0.89b	ND	ND	ND	128.86 ± 0.76b
Vanillin	ND	ND	ND	4.69 ± 0.32c	4.72 ± 0.07c	10.74 ± 0.21d
Taxifolin	30.07 ± 0.06a	36.19 ± 0.53b	ND	ND	422.99 ± 25.24c	ND

*Each value was expressed as mean ± standard deviation (n = 3). Values with different lowercase letters (a-e) within columns are significantly different (p < 0.05)*.

### Antioxidant Activities *In vitro*

In this article, various assays (DPPH^•^, ABTS^•^, FRAP, RP, and CUPRAC) were adopted to comprehensively evaluate the antioxidant activities of CIOP extracts. As shown in Table 4, three DESs extracts showed higher antioxidant abilities in DPPH^•^, ABTS^•^, FRAP, RP, and CUPRAC. Especially, ChCl-Xyl extracts exerted the strongest abilities in DPPH^•^ (109.00 μmol TE/g DW), ABTS^•^ (411.08 μmol TE/g DW), FRAP (1145.07 μmol TE/g DW), and RP (808.90 μmol TE/g DW) and CUPRAC (325.36 μmol TE/g DW), respectively. Better antioxidant activities were found in 50% MeOH extract as compared with water and EtAc extracts. EtAc extract had the lowest antioxidant activities. Remarkably, antioxidant activities of the CIOPs extracts varied with the type of solvent. The extracts with larger TPC/TFC had significantly better antioxidant activities. In addition, it can be found that ChCl-Xyl extract without the largest TPC/TFC exhibited the strongest antioxidant activities. It was probably due to the high contents of individual phenolics (arbutin, procyanidin B1, (+)-catechin, and (–)-epicatechin) in the ChCl-Xyl extracts led to strong antioxidant activities. Ma et al. (2021) reported that the polarity of solvent greatly affected the extraction yields and the compositions of phenolic compounds from Huangshan Gongju (a kind of chrysanthemum in China), thereby influencing the antioxidant activities of the extracts (36). A similar trend can be also observed by Wan et al. (37), who reported that the chemical compositions and antioxidant activity of *Chlorella vulgaris* extracts were greatly influenced by the extraction solvents. Simultaneously, Rafińska et al. (38) also found that the solvents had obvious effects on the phenolic profile and antioxidant capacity of *Moringa oleifera* leaves extracts.

**Table 4:** Antioxidant activities of the CIOPs extracts obtained by various solvents. Vehicle experiments of the corresponding solvents have been carried out.

**Extractions**	**DPPH^**•**^ (μmol TE/g DW)**	**ABTS^**+•**^ (μmol TE/g DW)**	**FRAP (μM Fe(II)E/g DW)**	**RP (μmol TE/g DW)**	**CUPRAC (μmol TE/g DW)**
H_2_O	47.75 ± 1.29b	214.54 ± 3.84b	233.60 ± 1.52b	203.71 ± 0.89b	33.97 ± 0.18b
50% MeOH	103.10 ± 0.42c	297.93 ± 1.53c	993.25 ± 1.93c	616.70 ± 0.29c	257.14 ± 2.34d
EtAc	0.34 ± 0.03a	4.25 ± 0.02a	9.05 ± 0.20a	4.77 ± 0.03a	15.11 ± 0.15a
ChCl-Xyl	109.00 ± 0.84d	411.08 ± 22.46e	1145.07 ± 6.58e	897.59 ± 30.06f	325.36 ± 1.67c
ChCl-Gly	106.47 ± 0.84c	317.40 ± 4.58d	1029.93 ± 3.29d	793.67 ± 5.31d	300.64 ± 5.71c
Bet-CA	105.07 ± 0.49c	302.48 ± 1.97c	1026.65 ± 8.70d	824.12 ± 3.20e	322.25 ± 0.31e

*Each value was expressed as mean ± standard deviation (n = 3). Values with different lowercase letters (a-f) within columns are significantly different (p <0.05)*.

### Inhibitory Activity Against α-Glucosidase

As we know, α-glucosidase can hydrolyze polysaccharides or starch into glucose or disaccharides, thereby increasing the blood sugar level. Consequently, suppressing the α-glucosidase activity in the small intestine can effectively control the postprandial blood sugar level. At present, the natural α-glucosidase inhibitors from natural products have attracted increasing interest in the management of high blood sugar (39, 40).

**Figure 3:**
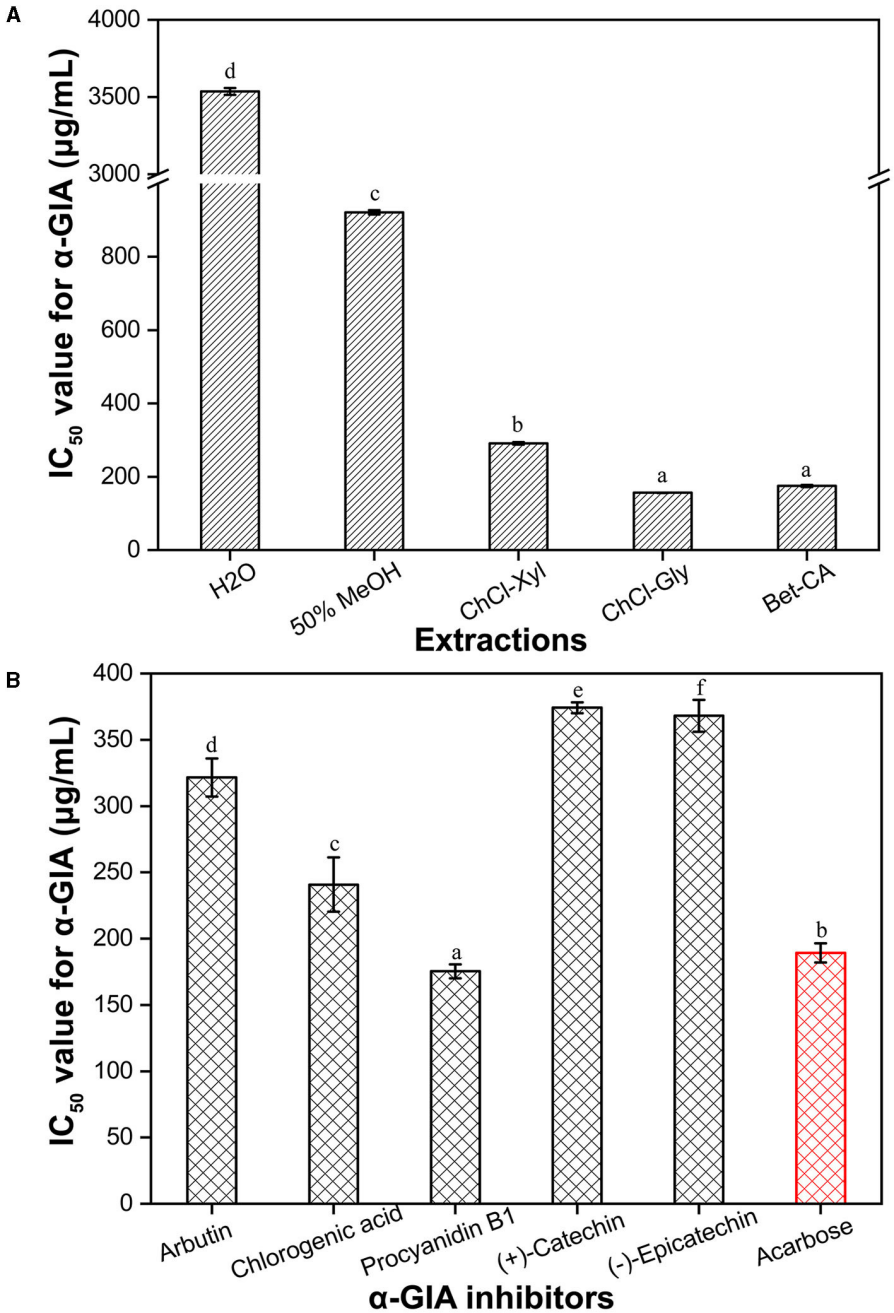
IC_50_ values for α-glucosidase inhibitory activity (α-GIA) of the CIOPs extracts **(A)** and the main phenolic compounds **(B)** in the extractions. Ethyl acetate (EtAc) extracts, no inhibitory activity. Different lowercase letters (a–e) mean statistically significant differences.

To comprehensively assess the anti-hypoglycemic effects *in vitro* of CIOPs, the α-GIA of the extracts was investigated (Figure 3A). It can be seen that the α-GIA of traditional solvents extracts (water, MeOH, and EtAc) was significantly lower than that of three DESs extracts (ChCl-Xyl, ChCl-Gly, and Bet-CA). Compared with the positive control acarbose (IC_50_ = 195.37 μg/ml), the three DESs extracts showed the strong α-GIA (IC_50_, 156.25–291.11 μg/ml), especially for ChCl-Gly extract (IC_50_ = 156.25 μg/ml). Water extract also showed good α-GIA (IC_50_ = 3535.93 μg/ml), but EtAc extract with the lowest TPC/TFC did not exhibit ideal α-GIA. It can be observed that the extracts with higher TPC/TFC had stronger α-GIA, which was in line with the results of Zhu et al. (23). To confirm the main contributors to α-GIA in the CIOPs extracts, the α-GIA of the main individual phenolics was investigated (Figure 3B). The results verified that all the investigated phenolics showed strong α-GIA (IC_50_ <400 μg/ml), especially for procyanidin B1 (IC_50_ = 68.72 μg/ml) and chlorogenic acid (IC_50_ = 240.80 μg/ml). Arbutin (IC_50_ = 321.54 μg/ml), (–)-epicatechin (IC_50_ = 368.10 μg/ml), and (+)-catechin (IC_50_ = 374.23 μg/ml) indicated relatively low α-GIA compared with the positive drug acarbose (IC_50_ = 195.37 μg/ml). In addition, we found that DESs extracts with higher contents of these phenolics (especially for procyanidin B1 and chlorogenic acid) showed stronger α-GIA than the other solvent extracts. Many researchers have confirmed that procyanidin B1, chlorogenic acid, and catechin, all have significant anti-diabetic activity in the treatment of streptozotocin-induced diabetic rats (41, 42).

### *In Silico* Analysis

**Figure 4:**
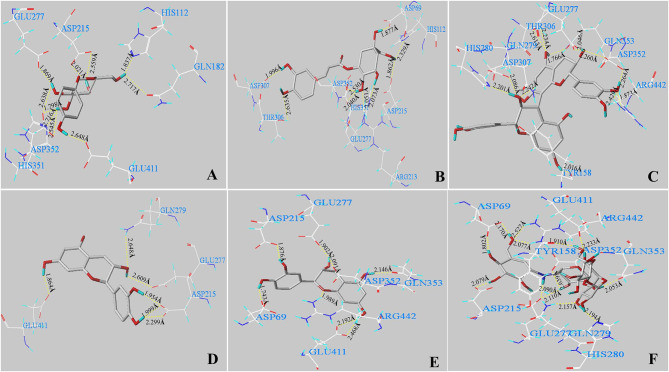
*In silico* analysis of the main phenolics/acarbose with α-glucosidase. The conformations of five main phenolics and acarbose interacting with amino acid residues in the active site of α-glucosidase: arbutin **(A)**, chlorogenic acid **(B)**, procyanidin B1 **(C)**, (+)-catechin **(D)**, (–)-epicatechin **(E)**, and acarbose **(F)** with residues in the active sites of the α-glucosidase, respectively. The dashed line represents hydrogen bonds.

*In silico* analysis was carried out to explain how the main phenolics [arbutin, chlorogenic acid, procyanidin B1, (+)-catechin, and (–)-epicatechin] in the CIOPs extracts might interact with α-glucosidase. Figures 4A–F indicates the 3D structures of five main phenolics/acarbose docking with α-glucosidase. In addition, all ligands could fit well into the binding pocket of the α-glucosidase homology model (Supplementary Figures 1A-F). Table 5 shows the generated docking information. It can be found that C-score values were ≥ 4 after all of the ligands were docked with the enzyme receptor. The T-score value of arbutin was 6.38, seven H-bonds were formed with 10 amino acid active residues of the α-glucosidase (ASP 215, ASP 352, ARG 213, GLN 182, GLU 277, GLU 411, HIS 112, and HIS 351). The distance of the H-bond ranged from 1.798 to 2.717 Å (Figure 4A; Table 5). Nine H-bonds (average distance of 2.155 Å) interactions with nine catalytic residues (ASP 69, ASP 215, ASP 307, ASP 352, ARG 213, GLU 277, HIS 112, HIS 351, and THR 306) were found in chlorogenic acid, with a relative high T-score of 8.78 (Figure 4B; Table 5). Procyanidin B1 with the lowest T-score of 6.99 indicated nine H-bonds interactions with 12 amino acid residues (ASP 307, ASP 352, ARG 442, GLN 279, GLN 353, GLU 277, HIS 280, THR 306, and TYR 158). The distance of H-bonds ranged from 1.766 to 2.613 Å (Figure 4C; Table 5). (+)-Catechin, with a T-score of 6.01, generated four H-bonds (average distance was 2.229 Å) with six catalytic residues (ASP 215, GLU 277, GLU 411, GLN 279) of the receptor (Figure 4D; Table 5). (–)-Epicatechin, with a T-score value of 5.66, indicated nine H-bonds (H-bond distance ranging from 1.743 to 2.468 Å) interactions with eight key amino acid residues of ASP 69, ASP 215, ASP 352, ARG 442, GLN 353, GLU 277, and GLU 411 of the receptor (Figure 4E; Table 5). As an anti-diabetic drug, acarbose with the highest T-score of 11.45 represented the extremely strong interactions with α-glucosidase and generated 10 H-bonds (average distance of 2.080 Å) interactions with 13 amino acid active residues (ASP 69, ASP 215, ASP 352, ARG 442, GLN 279, GLN 353, GLU 277, GLU 411, HIS 280, and TYR 158) of the α-glucosidase (Figure 4F; Table 5).

**Table 5:** Molecular interactions between the main phenolics/acarbose and α-glucosidase.

**Main phenolics**	**C-Score**	**T-Score**	***n* (binding residues)**	***n* (H-bond formation)**	**Active amino acid residues**
Arbutin	5	6.38	7	10	**ASP 215**, **ASP 352**, ARG 213, GLN 182, **GLU 277**, **GLU 411**, HIS 112, HIS 351
Chlorogenic acid	5	8.76	9	9	**ASP 69**, **ASP 215**, ASP 307, **ASP 352**, ARG 213, **GLU 277**, HIS 112, HIS 351, THR 306
Procyanidin B1	4	6.99	9	12	ASP 307, **ASP 352**, **ARG 442**, **GLN 279**, GLN 353, **GLU 277**, **HIS 280**, THR 306, TYR 158
(+)-Catechin	5	6.01	4	6	**ASP 215**, **GLU 277**, **GLU 411**, GLN 279
(–)-Epicatechin	4	5.66	7	8	**ASP 69**, **ASP 215**, **ASP 352**, **ARG 442**, GLN 353, **GLU 277**, **GLU 411**
Acarbose^42^	5	11.45	10	13	**ASP 69, ASP 215**, **ASP 352**, **ARG 442**, **GLN 279**, GLN 353, **GLU 277, GLU 411**, **HIS 280**, TYR 158

*The bold font indicates these key amino acid catalytic residues*.

Normally, the binding active sites of the ligands docked with the receptors can affect the catalytic ability of the enzyme. With regard to the main phenolics/acarbose docked with α-glucosidase, the numbers of binding sites ranked in the following order: acarbose (13) > procyanidin B1 (12) > arbutin (10) > chlorogenic acid (9) > (–)-epicatechin (8) > (+)-catechin (6); the number of formed H-bonds ranked as follows: acarbose (10) > chlorogenic acid (9) = procyanidin B1 (9) > (–)-epicatechin (7) = arbutin (7) > (+)-catechin (5). There was a significant positive relationship between the number of H-bonds and binding active sites residues with α-GIA. The ligands with a larger number of H-bonds and catalytic residues (acarbose, chlorogenic acid, and procyanidin B1) showed higher α-GIA, which was in line with the findings of Cai et al. (43). In addition, some studies confirmed that the key active sites of α-glucosidase (Asp 69, Asp 215, ASP 352, ARG 442, GLU 277, GLN 279, HIS 280, and Glu 411) exerted the catalytic ability for α-glucosidase (44). In this work, acarbose interacted with these key active sites of α-glucosidase, thereby indicating strong α-GIA. (+)-Catechin had the least H-bonds and catalytic residues and cannot interact with some major catalytic sites (ASP 69, ASP 352, ARG 442, and GLU 277) of α-glucosidase, so it had the worst α-GIA. Cai et al. (2021) confirmed that the active catalytic sites (Asp 69, Asp 215, ASP 352, GLU 277, HIS 280, and Glu 411) of the ligands interacted with the α-glucosidase receptors greatly affected α-GIA (43).

### Multivariate Analysis

**Figure 5:**
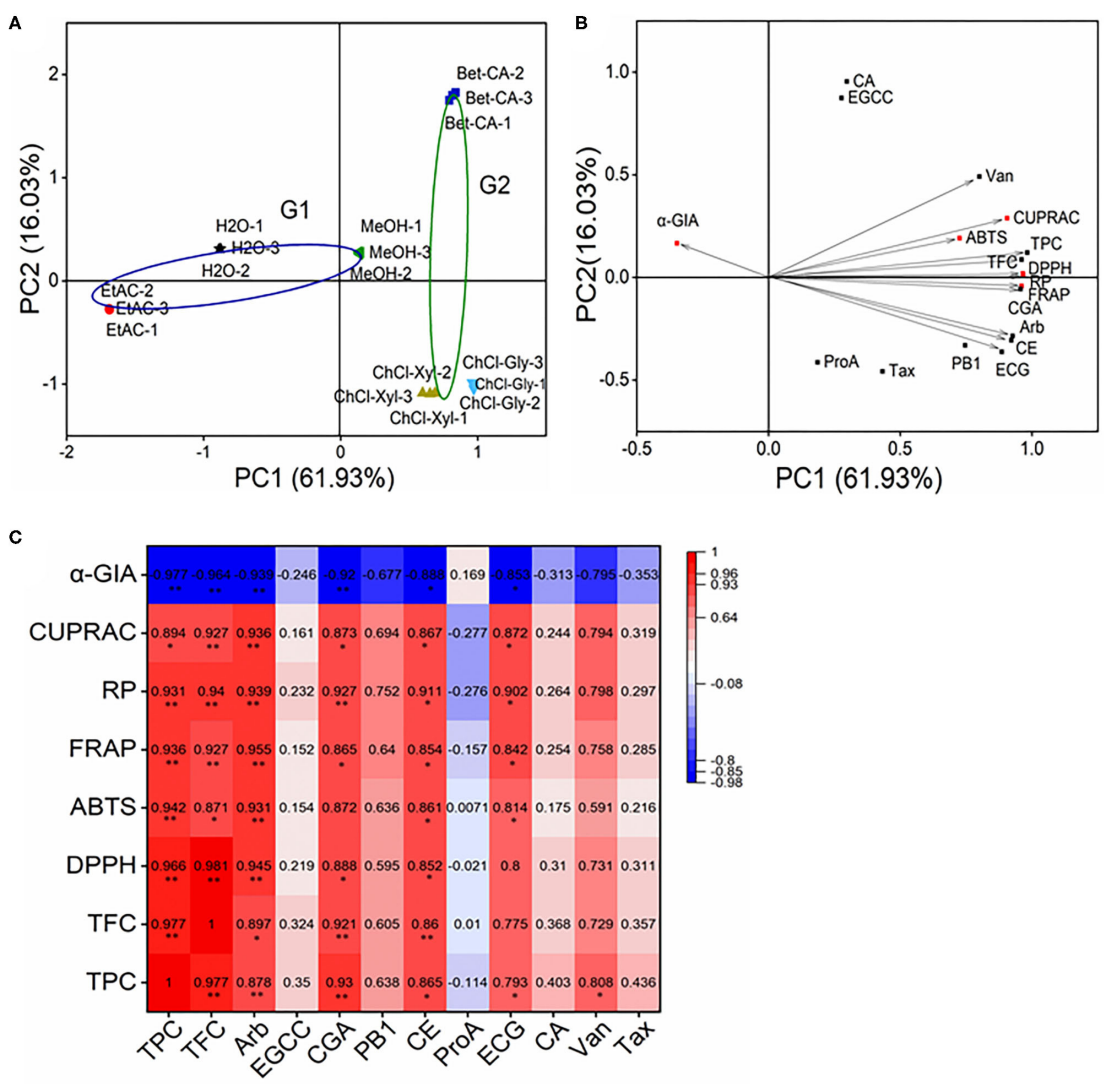
Principal component analysis (PCA) and correlation matrix of CIOPs extracts. **(A)**, PCA score plot; **(B)**: PCA loading plot. **(C)**: Heatmap analysis of correlation matrix. Arb, arbutin; EGCC, (–)-epigallocatechin; CGA, chlorogenic acid; PB1, procyanidin B1; CE, (+)-catechin; ProA, protocatechuic acid; ECG, epicatechin; CA, caffeic acid; Van, vanillin; Tax, taxifolin.

Principal component analysis (PCA) was carried to visualize the impact of solvent on phenolic constituents and the bio-activities of the CIOPs extracts. PC1 61.93% and PC2 16.03% took up 77.96% of the total variances, which indicated that these two principal components could load maximum information of the original data. For the PCA loading plot, the traditional solvents and DESs were, respectively, divided into G1 and G2 (Figure 5A). With respect to the PCA score plot, the relationship between samples can be represented by the distance between the points, and the relationship between the variables can be reflected by the cosine values (Figure 5B), the small distance or cosine values of two loadings indicated these had a good correlation. Among them, TPC, TFC, arbutin (Arb), vanillin (Van), chlorogenic acid (CGA), procyanidin B1 (PB1), (+)-catechin (CE), epicatechin (EC), and vanillin (Van) were extremely correlated with DPPH^•^, ABTS^+•^, FRAP, RP, and CUPRAC. In addition, TPC, TFC, Arb, CGA, PB1, CE, and EC were strongly correlated with α-GIA. The PCA results verified that the extraction solvents had a great impact on the biological activities of the CIOPs extracts.

A heatmap analysis can better visualize the coherent matrix between the phenolic constituents and the bio-activities of the CIOPs extracts (Figure 5C). A noteworthy positive correlation was observed among TPC, TFC, DPPH^•^, ABTS^+•^, FRAP, RP, CUPRAC, and α-GIA. The results were as follows: TFC vs. TPC (*r* = 0.977, *p* < 0.01), TFC vs. DPPH^•^ (*r* = 0.981, *p* < 0.01), TFC vs. ABTS^+•^ (*r* = 0.871, *p* < 0.05), TFC vs. FRAP (*r* = 0.927, *p* < 0.01), TFC vs. RP (*r* = 0.940, *p* < 0.01), TFC vs. CUPRAC (*r* = 0.927, *p* < 0.01), TFC vs. α-GIA (*r* = −0.964, *p* < 0.01); TPC vs. DPPH^•^ (*r* = 0.966, *p* < 0.01), TPC vs. ABTS^+•^(*r* = 0.942, *p* < 0.01), TPC vs. FRAP (*r* = 0.936, *p* < 0.01), TPC vs. RP (*r* = 0.931, *p* < 0.01), TPC vs. CUPRAC (*r* = 0.894, *p* < 0.05), TPC vs. α-GIA (*r* = −0.977, *p* < 0.01). Remarkably, Arb and CE showed strong correlations with the DPPH^•^, ABTS^+•^, FRAP, RP, and CUPRAC; CGA showed strong correlations with the DPPH^•^, FRAP, RP, and CUPRAC; ECG was strongly correlated with the ABTS^+•^, FRAP, RP, and CUPRAC, which agreed well with the previous study (45, 46). Arb, CGA, CE, and ECG were strongly correlated with α-GIA. Phenolic compounds from natural products, such as arbutin, chlorogenic acid, (+)-catechin, and (–)-epicatechin, have been previously reported to have strong antioxidant activities and α-GIA, which have been extensively used in the food and pharmacy industries (47). A multivariate analysis also verified the main contributors in the CIOPs extracts to antioxidant activities (Arb, CGA, VAN, CE, and ECG) and α-GIA (Arb, CGA, PB1, CE, and ECG).

## Conclusion

*Clausena indica* (Datz.) Oliv fruit pericarps extracts extracted with the traditional solvents and eco-friendly solvents showed significant differences in the phenolic profiles, antioxidant activities, and α-GIA. Phenolic compositions of the CIOPs extracts were identified for the first time using LC-Q-Orbitrap-MS/MS. Arbutin, (–)-epigallocatechin, chlorogenic acid, procyanidin B1, (+)-catechin, and (–)-epicatechin were dominant components in the extracts, especially for the DESs extracts. In addition, ChCl-Xyl and ChCl-Gly extracts showed more excellent antioxidant activities than other solvents extracts. Three DESs extracts with higher TPC and TFC (especially for arbutin, chlorogenic acid, and procyanidin B1) indicated stronger α-GIA. Furthermore, *in silico* analysis was carried out to determine the α-glucosidase-inhibiting mechanisms of the main phenolics. Multivariate analysis also testified the main contributors in the CIOPs extracts to antioxidant activities and α-GIA. In conclusion, DES can be considered as a promising eco-friendly solvent for the valorization and recovery of high-value compounds from agro-industrial by-products. Furthermore, CIOPs can be applied as a prospective source of active compounds applied in the food and pharmaceutical industries.

## Data Availability Statement

The original contributions presented in the study are included in the article/Supplementary Material, further inquiries can be directed to the corresponding author/s.

## Author Contributions

RW: conceptualization. RW and RH: data curation, methodology, and software. LW: funding acquisition, project administration, supervision, and writing—review and editing. RW, RH, and ZL: investigation. RH and ZL: resources. RH and RW: software. ZL: validation and writing—original draft. All authors contributed to the article and approved the submitted version.

## Conflict of Interest

The authors declare that the research was conducted in the absence of any commercial or financial relationships that could be construed as a potential conflict of interest.

## Publisher's Note

All claims expressed in this article are solely those of the authors and do not necessarily represent those of their affiliated organizations, or those of the publisher, the editors and the reviewers. Any product that may be evaluated in this article, or claim that may be made by its manufacturer, is not guaranteed or endorsed by the publisher.

## Supplementary Material

Supplementary Figure 1 3D docking structures of the seven potential inhibitors and acarbose were inserted into the hydrophobic cavity α-glucosidase by molecular docking analysis: arbutin **(A)**, chlorogenic acid **(B)**, procyanidin B1 **(C)**, (+)-catechin **(D)**, (–)-epicatechin **(E)**, and acarbose **(F)**. The dashed line represents hydrogen bonds.

Supplementary Table 1 Regression equation, R^2^, Linear range results of the main analytes.
