# Supplementary figures and images for: LC-Q-Orbitrap-MS/MS Characterization, Antioxidant Activity, and α-Glucosidase-Inhibiting Activity With In Silico Analysis of Extract From Clausena Indica (Datz.) Oliv Fruit Pericarps
Source: Front Nutr. 2021 Sep 1;8:727087. doi: 10.3389/fnut.2021.727087 (PMC8440871; doi:10.3389/fnut.2021.727087)

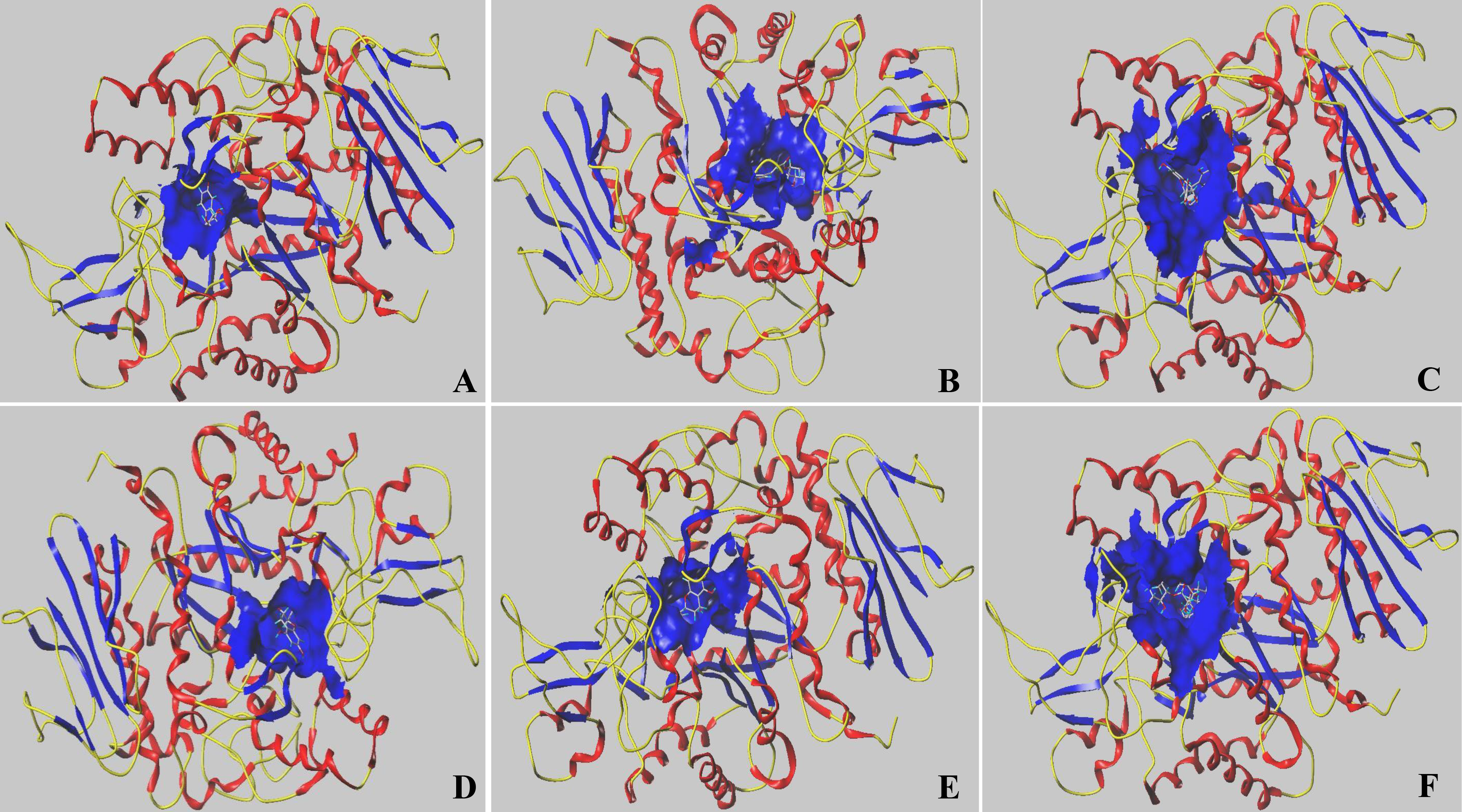

Supplement: Supplementary Figure 1 — 3D docking structures of the seven potential inhibitors and acarbose were inserted into the hydrophobic cavity α-glucosidase by molecular docking analysis: arbutin (A), chlorogenic acid (B), procyanidin B1 (C), (+)-catechin (D), (–)-epicatechin (E), and acarbose (F). The dashed line represents hydrogen bonds. [file Image_1.JPEG]
