# Supplementary material for: LC-Q-Orbitrap-MS/MS Characterization, Antioxidant Activity, and α-Glucosidase-Inhibiting Activity With In Silico Analysis of Extract From Clausena Indica (Datz.) Oliv Fruit Pericarps
Source: Front Nutr. 2021 Sep 1;8:727087. doi: 10.3389/fnut.2021.727087 (PMC8440871; doi:10.3389/fnut.2021.727087)
Supplement: Supplementary Table 1 — Regression equation, R2, Linear range results of the main analytes. [file Table_1.DOC]

**Table S1**

Regression equation, R^2^, Linear range results of the main analytes

| Analytes | Calibration curve | R^2^ | Linear range (μg/mL) |
| --- | --- | --- | --- |
| Arbutin | Y = 13.843X - 63.005 | 0.9919 | 10-100 |
| (-)-Epigallocatechin | Y = 0.9553X + 27.913 | 0.9950 | 20-200 |
| Chlorogenic acid | Y = 19.284X - 18.364 | 0.9947 | 10-100 |
| Procyanidin B1 | Y = 6.8394X - 195.43 | 0.9929 | 10-100 |
| (+)-Catechin | Y = 5.0548X - 25.219 | 0.9983 | 10-100 |
| Protocatechuic acid | Y = 124.5X - 69.51 | 0.9991 | 10-100 |
| (-)-Epicatechin | Y = 23.916X - 83.551 | 0.9944 | 10-100 |
| Caffeic acid | Y = 84.967X - 147.83 | 0.9980 | 10-100 |
| Vanillin | Y = 61.537X - 91.412 | 0.9966 | 10-100 |
| Taxifolin | Y = 26.458X - 59.372 | 0.9949 | 10-100 |
